# Supplementary figures and images for: Beta 1 integrin signaling mediates pancreatic ductal adenocarcinoma resistance to MEK inhibition
Source: Sci Rep. 2020 Jul 7;10:11133. doi: 10.1038/s41598-020-67814-9 (PMC7340786; doi:10.1038/s41598-020-67814-9)

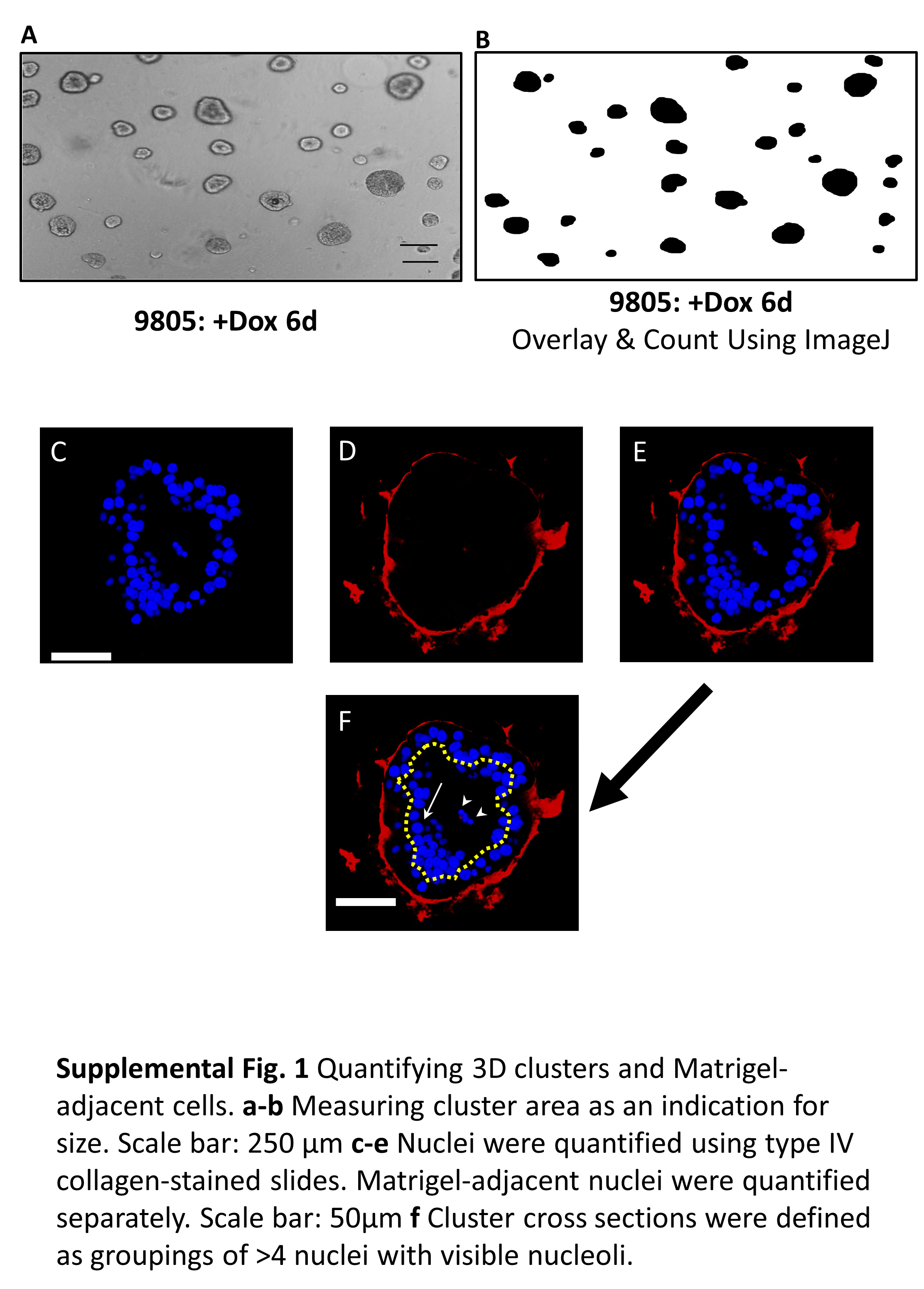

Supplement: Supplementary file 1 — Supplementary Figure 1 [file 41598_2020_67814_MOESM1_ESM.tif]

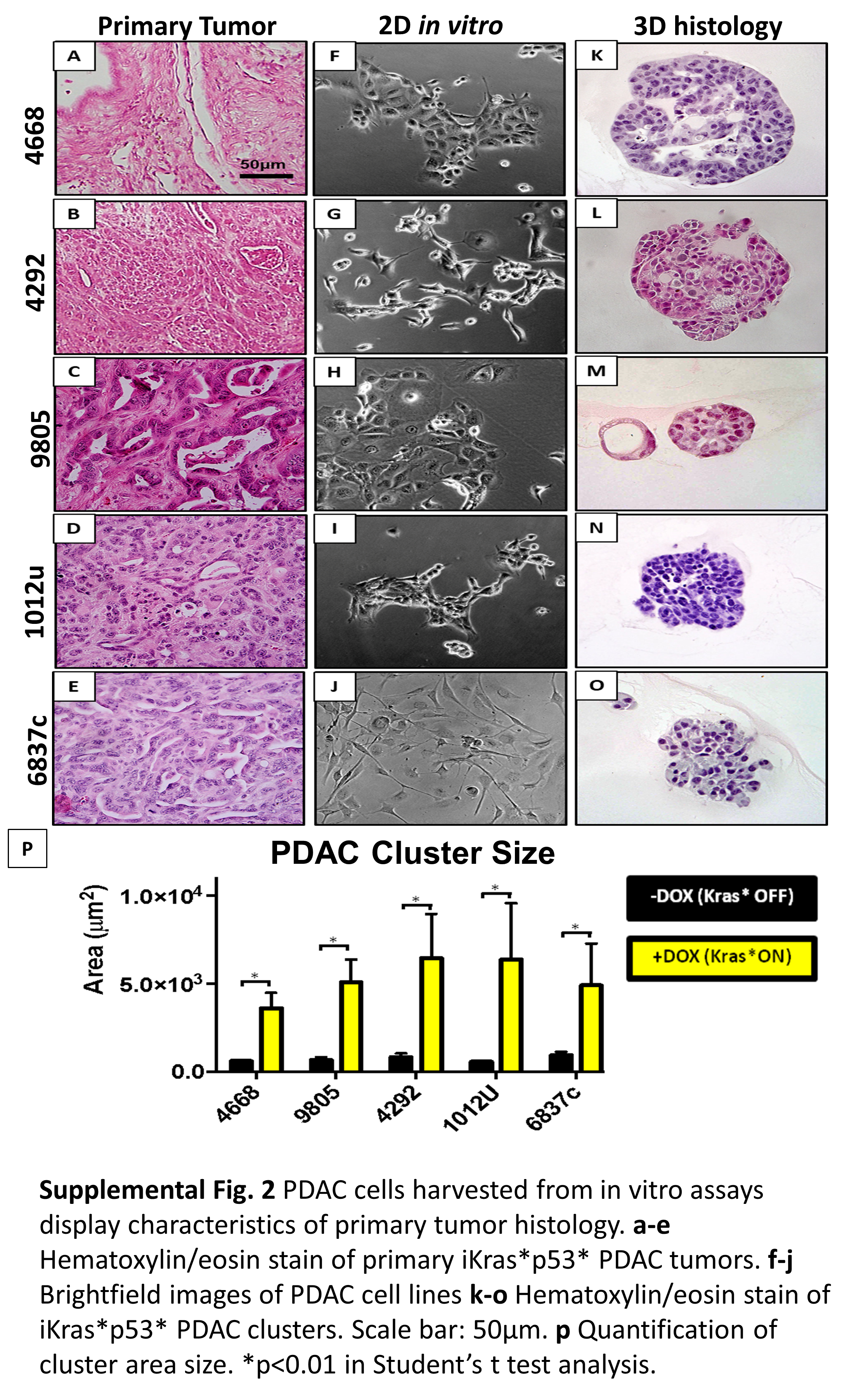

Supplement: Supplementary file 2 — Supplementary Figure 2 [file 41598_2020_67814_MOESM2_ESM.tif]

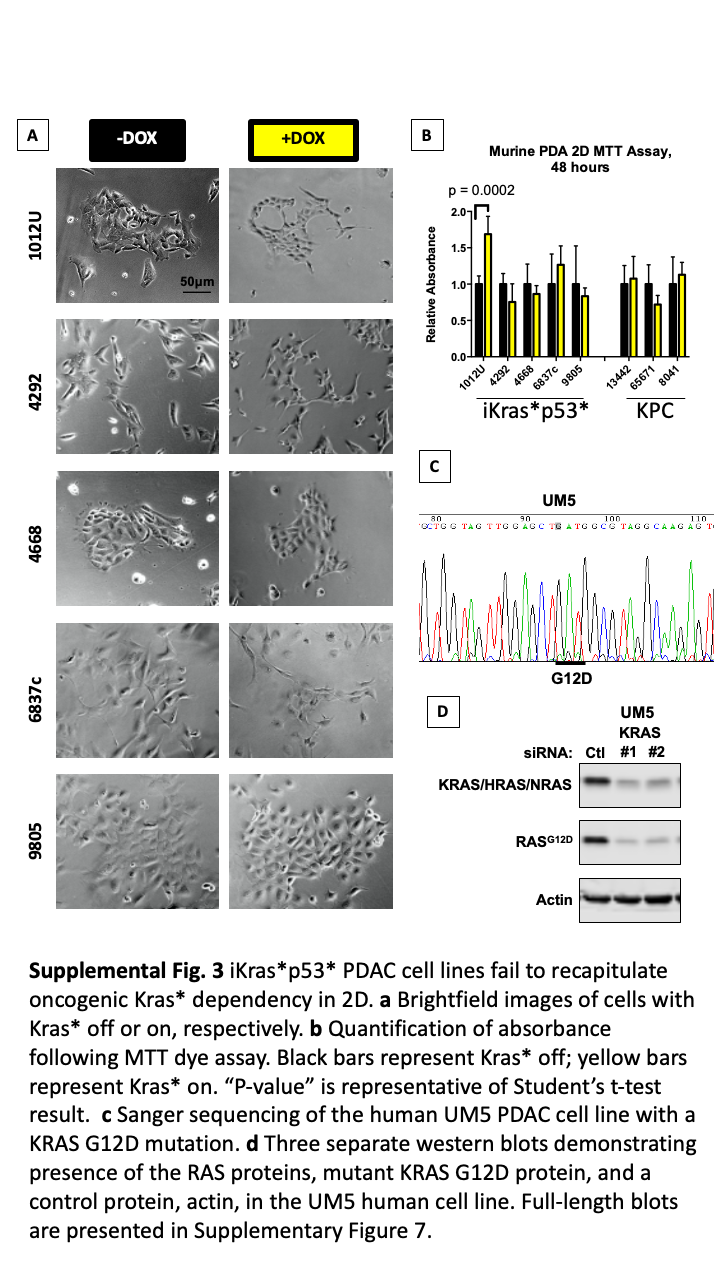

Supplement: Supplementary file 3 — Supplementary Figure 3 [file 41598_2020_67814_MOESM3_ESM.tiff]

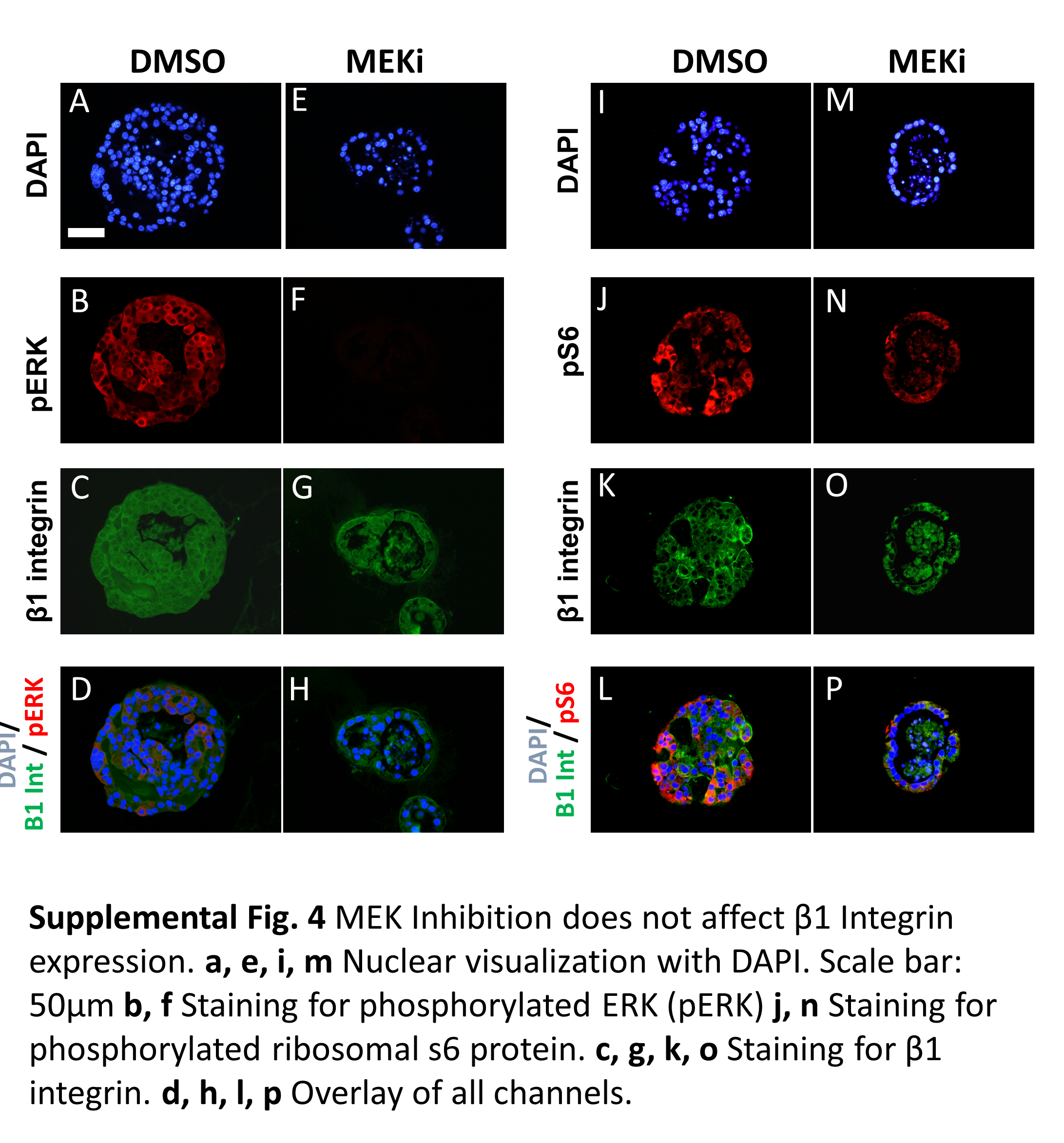

Supplement: Supplementary file 4 — Supplementary Figure 4 [file 41598_2020_67814_MOESM4_ESM.tif]

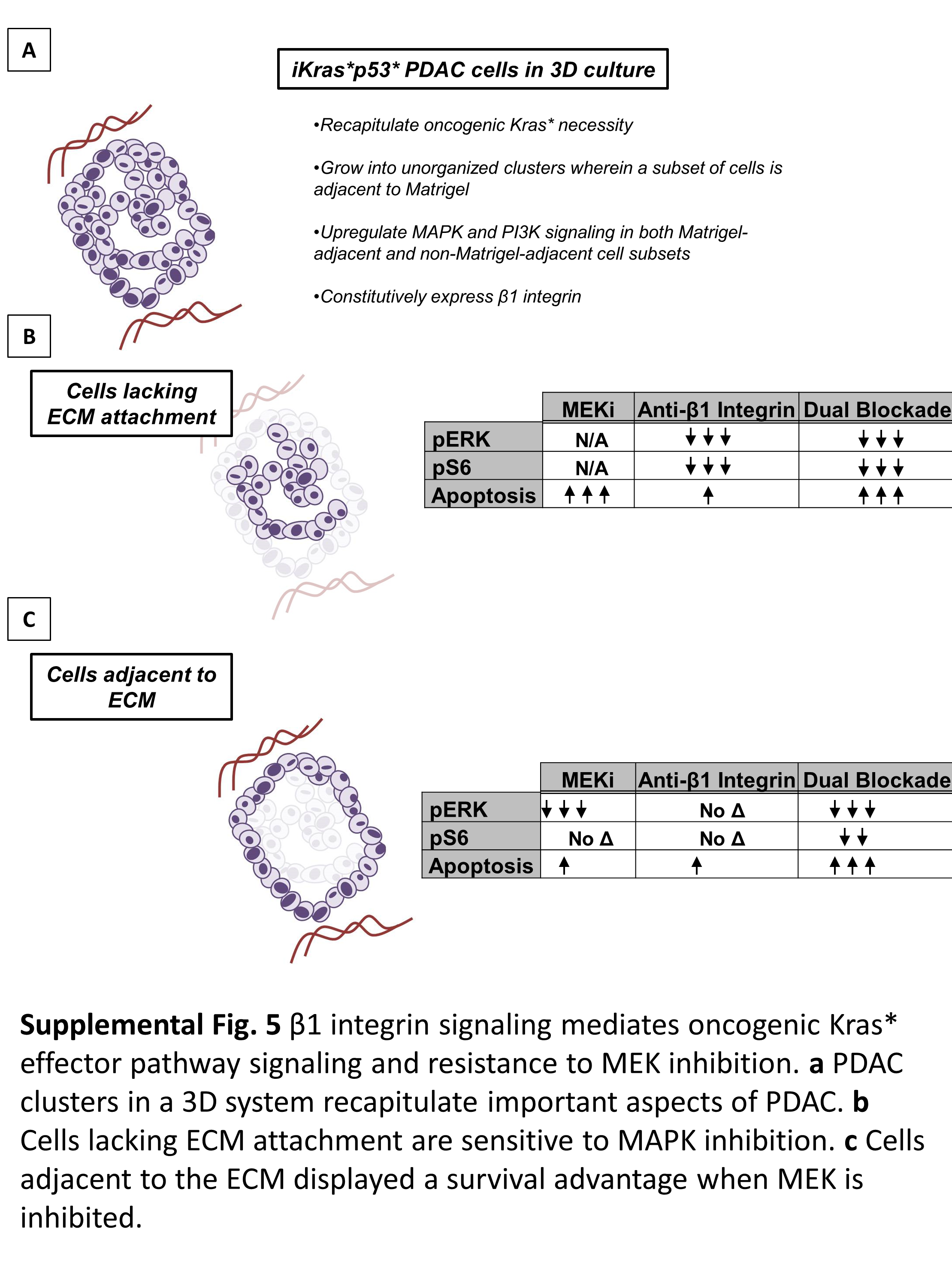

Supplement: Supplementary file 5 — Supplementary Figure 5 [file 41598_2020_67814_MOESM5_ESM.tif]

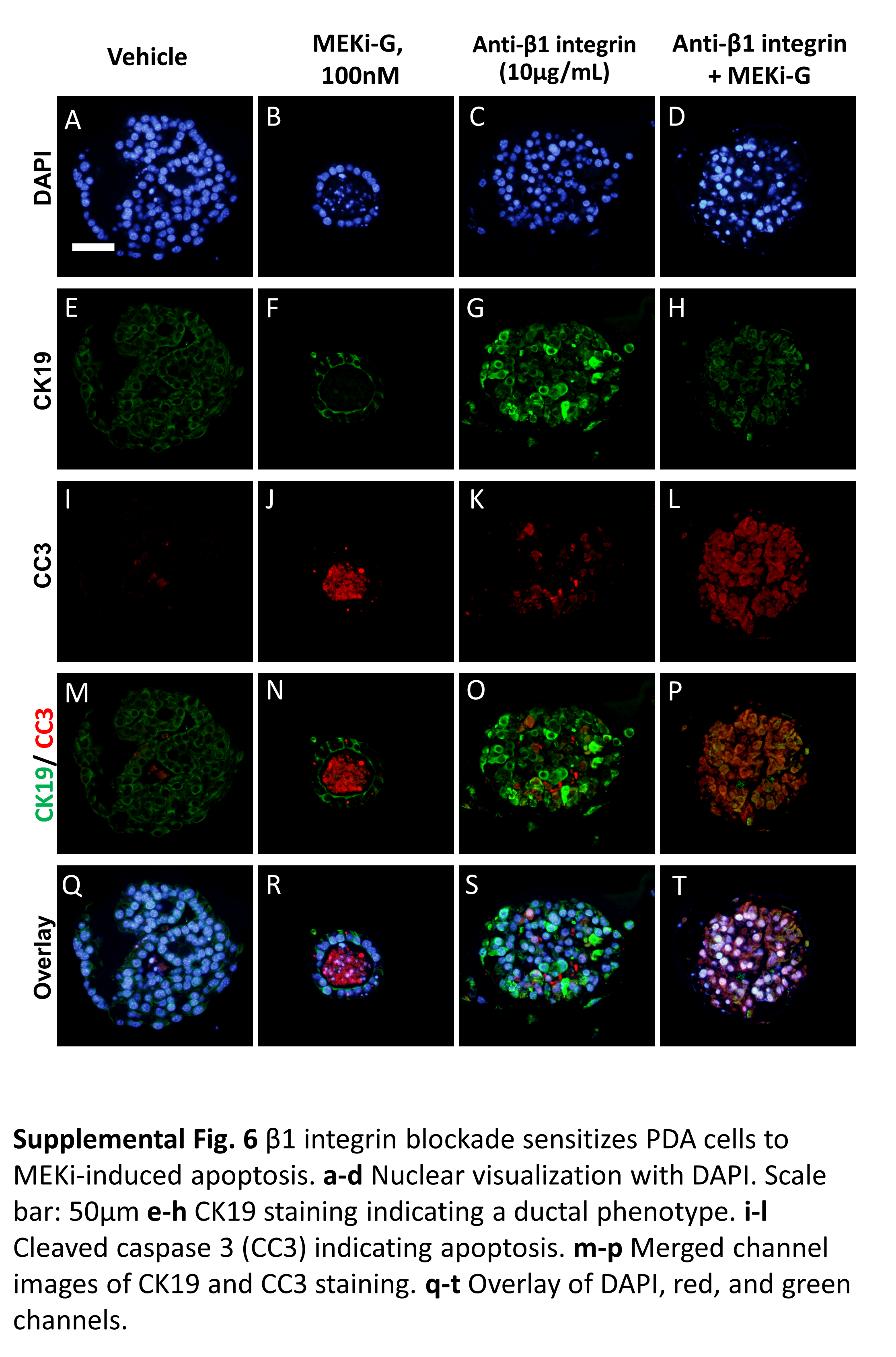

Supplement: Supplementary file 6 — Supplementary Figure 6 [file 41598_2020_67814_MOESM6_ESM.tif]

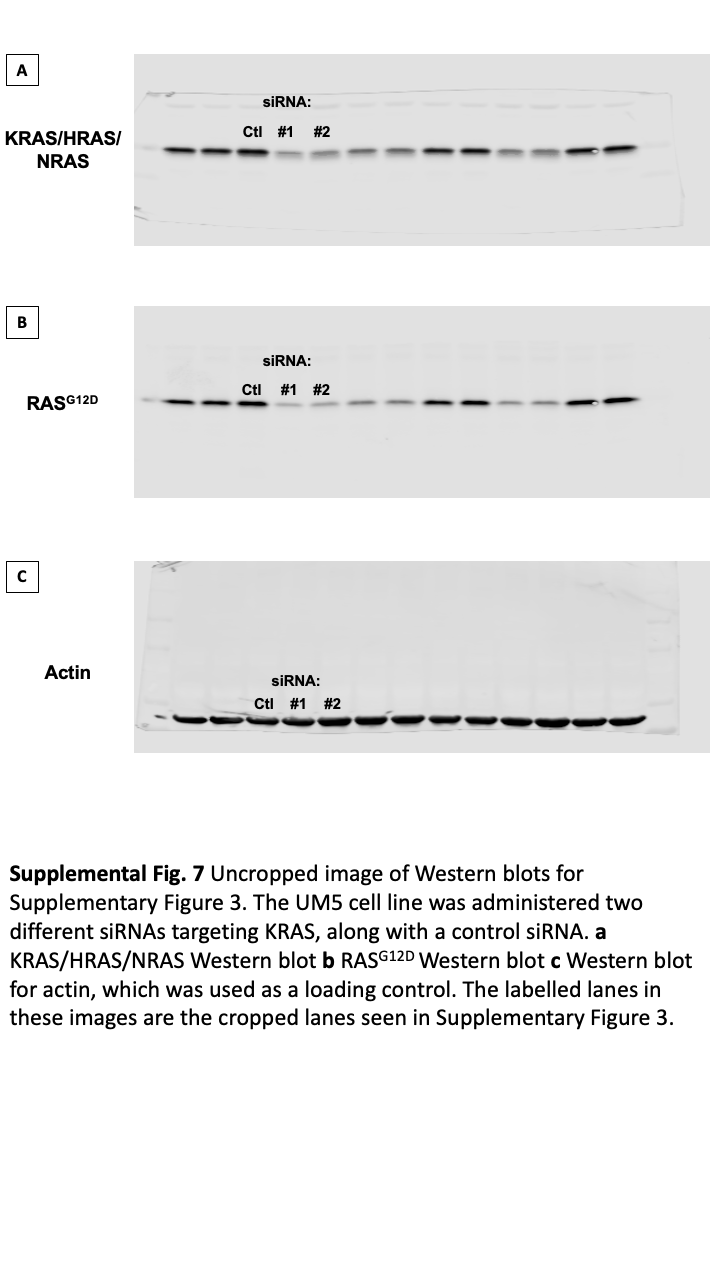

Supplement: Supplementary file 7 — Supplementary Figure 7 [file 41598_2020_67814_MOESM7_ESM.tiff]

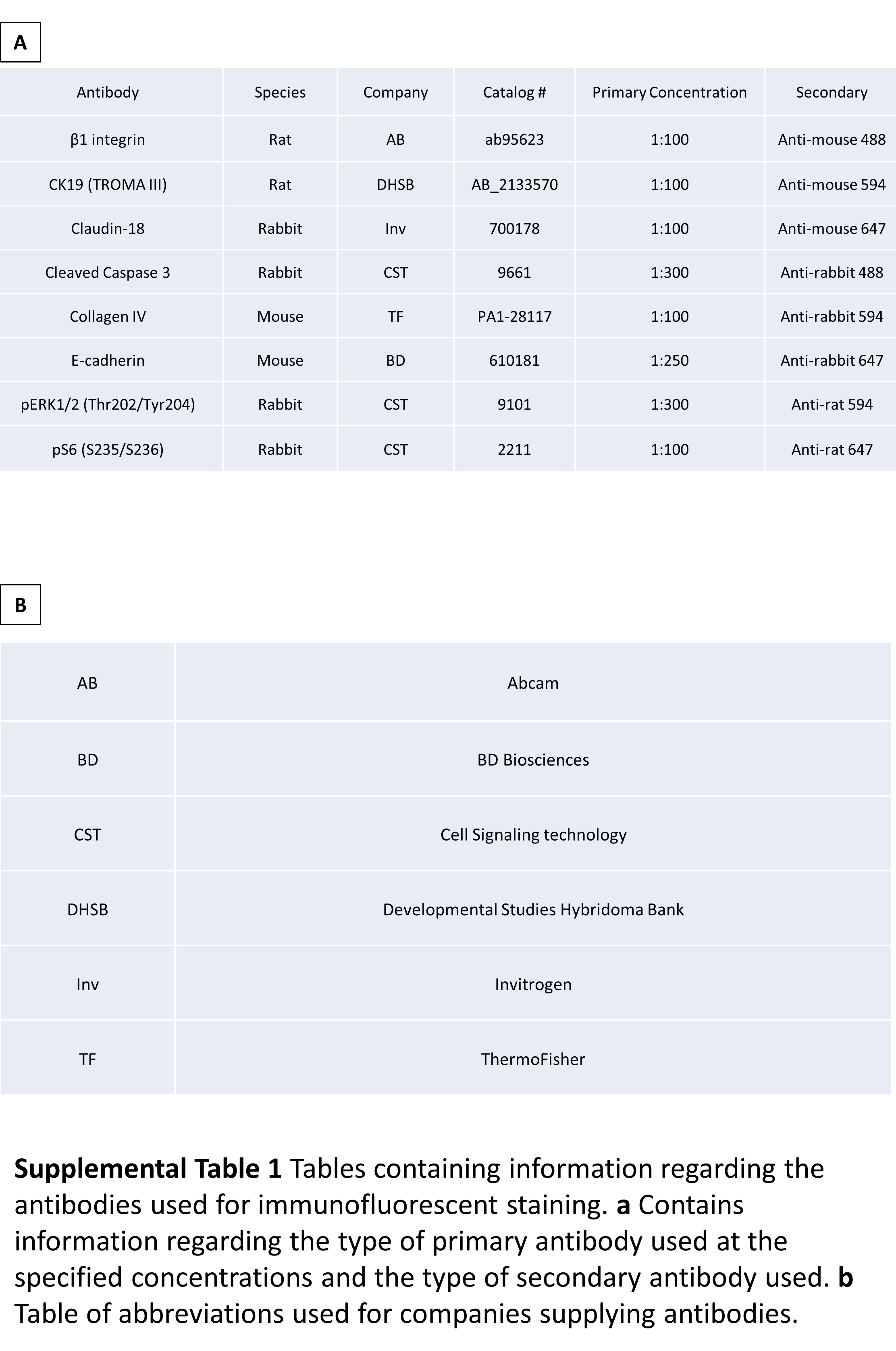

Supplement: Supplementary file 8 — Supplementary Table 1 [file 41598_2020_67814_MOESM8_ESM.tif]
